# Early Steps of HIV-1 Fusion Define the Sensitivity to Inhibitory Peptides That Block 6-Helix Bundle Formation

**DOI:** 10.1371/journal.ppat.1000585

**Published:** 2009-09-18

**Authors:** Kosuke Miyauchi, Michael M. Kozlov, Gregory B. Melikyan

**Affiliations:** 1 Institute of Human Virology and Department of Microbiology and Immunology, University of Maryland School of Medicine, Baltimore, Maryland, United States of America; 2 Department of Physiology and Pharmacology, Sackler Faculty of Medicine, Tel Aviv University, Tel Aviv, Israel; Harvard Medical School, United States of America

## Abstract

The HIV envelope (Env) glycoprotein mediates membrane fusion through sequential interactions with CD4 and coreceptors, followed by the refolding of the transmembrane gp41 subunit into the stable 6-helix bundle (6HB) conformation. Synthetic peptides derived from the gp41 C-terminal heptad repeat domain (C-peptides) potently inhibit fusion by binding to the gp41 pre-bundle intermediates and blocking their conversion into the 6HB. Our recent work revealed that HIV-1 enters cells by fusing with endosomes, but not with the plasma membrane. These studies also showed that, for the large part, gp41 pre-bundles progress toward 6HBs in endosomal compartments and are thus protected from external fusion inhibitors. Here, we examined the consequences of endocytic entry on the gp41 pre-bundle exposure and on the virus' sensitivity to C-peptides. The rates of CD4 and coreceptor binding, as well as the rate of productive receptor-mediated endocytosis, were measured by adding specific inhibitors of these steps at varied times of virus-cell incubation. Following the CD4 binding, CCR5-tropic viruses recruited a requisite number of coreceptors much faster than CXCR4-tropic viruses. The rate of subsequent uptake of ternary Env-CD4-coreceptor complexes did not correlate with the kinetics of coreceptor engagement. These measurements combined with kinetic analyses enabled the determination of the lifetime of pre-bundle intermediates on the cell surface. Overall, these lifetimes correlated with the inhibitory potency of C-peptides. On the other hand, the basal sensitivity to peptides varied considerably among diverse HIV-1 isolates and ranked similarly with their susceptibility to inactivation by soluble CD4. We conclude that both the longevity of gp41 intermediates and the extent of irreversible conformational changes in Env upon CD4 binding determine the antiviral potency of C-peptides.

## Author Summary

The human immunodeficiency virus (HIV) envelope glycoprotein (Env) mediates fusion between the viral and cell membranes. The fusion is initiated by Env-receptor interactions and is followed by coreceptor binding and refolding of the transmembrane gp41 subunit. The gp41 refolding proceeds through several distinct intermediates, culminating in the formation of a final helical bundle structure which is blocked by inhibitory peptides targeting the complementary domains of gp41. We have recently shown that the exposure time of gp41 intermediates on the cell surface is limited by productive HIV endocytosis leading to fusion with endosomes. Here, we measured the rates of progression of different HIV isolates through distinct intermediate steps accessible to fusion inhibitors and correlated these rates with the inhibitory potency of peptides against these viruses. Whereas the potency of peptides was proportional to the lifetime of gp41 intermediates on the cell surface, the baseline sensitivity of the virus was also Env context-dependent. Higher concentrations of these inhibitors were required to block fusion induced by glycoproteins that were more resistant to inactivation by the soluble receptor. Collectively, these findings imply that both the kinetic factors and the stability of Env-receptor complexes control the HIV sensitivity to inhibitory peptides.

## Introduction

HIV Env-induced fusion between the viral and cellular membrane progresses through a series of steps that begin with binding of the gp120 subunit to CD4. This step results in the formation of the gp120 bridging sheet which, along with the third hypervariable loop (V3 loop), forms the coreceptor binding site (reviewed in [1]). The recruitment of coreceptors, CCR5 or CXCR4, by Env-CD4 complexes initiates gp41 refolding that progresses through a pre-bundle intermediate, in which the gp41 N- and C-terminal heptad repeat domains (N-HR and C-HR, respectively) are exposed [2]–[5]. The heptad repeat domains ultimately coalesce into the stable post-fusion conformation referred to as the 6-helix bundle (6HB). The 6HB is formed by an antiparallel association of the trimeric N-HR domain (coiled coil) with three peripheral C-HR domains (reviewed in [6]). In a pre-bundle conformation, gp41 is susceptible to inhibition by synthetic peptides derived from its C-HR domain (hereafter referred to as C-peptides). These peptides bind to the complementary N-HR region and block HIV fusion by preventing the formation of 6HBs [6]–[8].

The kinetics of HIV fusion and the progression of gp41 pre-bundles to the 6HB has been studied in a cell-cell fusion model [4], [9]–[13]. Biochemical studies using a tagged C-peptide showed that, depending on the virus strain, the gp41 coiled coils can be exposed as early as upon CD4 binding [2]. Once formed, the pre-bundles are thought to persist for a couple of minutes prior to converting into the 6HB [14]. Using a real-time cell-cell fusion assay, we observed that small fusion pores collapsed soon after the addition of C-peptides [13], indicating that the formation of 6HBs was not completed at this point. These findings show that Env remains vulnerable to inhibitors of 6HB formation and to antibodies targeting gp41 intermediates throughout the fusion reaction.

Our recent work revealed important differences between cell-cell and virus-cell fusion models [15]. Whereas HIV-1 Env can mediate cell fusion by merging two plasma membranes, the virus itself fails to release its content at the cell surface. Instead, HIV-1 fuses with endosomes, presumably after undergoing CD4- and coreceptor-mediated endocytosis. Time-resolved single virus imaging showed that fusion with the plasma membrane was blocked at a stage downstream of lipid mixing and did not progress to productive entry. Importantly, endosomal fusion was markedly delayed relative to virus internalization, demonstrating that the surface exposure of gp41 intermediates is limited by the relatively quick virus clearance from the cell surface. Endocytic HIV-1 entry could thus attenuate the effects of neutralizing antibodies and C-peptides that target intermediate conformations of Env.

Disparate HIV isolates are known to exhibit a broad range of sensitivities to C-peptides (e.g., [16]), but the mechanisms underlying this differential sensitivity are not well understood. Functional studies suggested a correlation between the potency of a 34-residue long peptide, C34, and the propensity of Env to expose the gp41 coiled coil domains upon binding to a soluble CD4 (sCD4) [2],[17],[18]. The efficacy of C-peptides is also modulated by their primary sequence and the sequence of complementary N-HR domains that determine the binding affinity [19],[20]. However, the potency of C34 peptides derived from HIV-1, HIV-2 and SIV isolates poorly correlated with their propensity to form stable 6HBs with the complementary N-HR domains [17].

Another determinant of the efficacy of C-peptides is thought to be the window of opportunity for their binding to gp41 intermediates. This notion is based on correlation between the kinetics of cell-cell fusion and the HIV-1 resistance to C-peptides [9],[10],[17],[18],[21]. These findings led to a hypothesis that the gp41 residence time in pre-bundle conformations determines the HIV-1 sensitivity to C-peptides [9]. Implicit for this model is the inverse relationship between the rate of fusion and the longevity of pre-bundles and the slow, rate-limiting binding of C-peptides to these intermediate conformations. The slow peptide binding would require prolonged exposure of the gp41 coiled coil and would thus limit its ability to block the quickly-progressing fusion. However, since the HIV-1 fusion proceeds through intermediate steps at which the gp41 coiled coils are not exposed, the overall kinetics of fusion may not reflect the window of opportunity for the peptide binding. Thus, in order to meaningfully examine the kinetic determinants of the HIV resistance to C-peptides, one needs to determine the actual residence time of gp41 in pre-bundle conformations on the cell surface.

Endocytic entry of HIV-1 warrants careful examination of the relationship between the rates of the surface-accessible pre-fusion steps and the sensitivity to C-peptides. Here, we employed inhibitors of distinct steps of HIV-1 fusion to monitor the progression through CD4 and coreceptor binding steps followed by productive endocytosis that protected the virus from fusion inhibitors employed in this study. Using a simple kinetic model of HIV fusion, we determined the rates of HIV-1 progression through key pre-fusion intermediates and thus the residence times of Env in these intermediates. Our results imply that multiple factors contribute to the potency of C-peptides. An important kinetic factor is the lifetime of gp41 pre-bundles which is defined by the rate of engagement of a requisite number of receptors and coreceptors on one hand and the productive virus endocytosis on the other. We also found that viral determinants, such as the extent of conformational changes in Env in response to the CD4 binding, significantly modulate the susceptibility of HIV-1 to C-peptides. The ability to evaluate the HIV-1 residence time in intermediate states permits rationalization of the complex mechanisms that define the resistance to C-peptides and other inhibitors targeting intermediate conformations of Env.

## Results

### Productive HIV-1 entry occurs through sequential CD4 and coreceptor binding followed by virus endocytosis

In order to directly monitor HIV-1 fusion with target cells, we measured the cytosolic activity of the beta-lactamase-Vpr (BlaM-Vpr) chimera packaged into the viral core [22]. Pseudoviruses containing the reporter enzyme were bound to target cells in the cold, and their fusion was initiated by quickly raising the temperature to 37°C, as described in [15]. The BlaM activity originates exclusively from viral cores delivered into the cytosol through fusion, whereas cell-bound or internalized viruses do not contribute to the signal [22]. Due to the low number of fused viruses, an overnight incubation is required to accumulate detectable amounts of cleaved fluorogenic BlaM substrate loaded into the cytosol. In order to resolve the kinetics of HIV fusion that occurs within a few hours (e.g., [23]), high concentrations of fusion inhibitors were added at varied times of incubation at 37°C [15]. The acquisition of resistance to a membrane-impermeant inhibitor yields the kinetics of virus progression beyond the step targeted by that inhibitor. Alternatively, resistance to inhibitors targeting a late step of fusion can occur through virus internalization that protects it from external inhibitors and permits subsequent fusion with endosomes.

**Figure 1:**
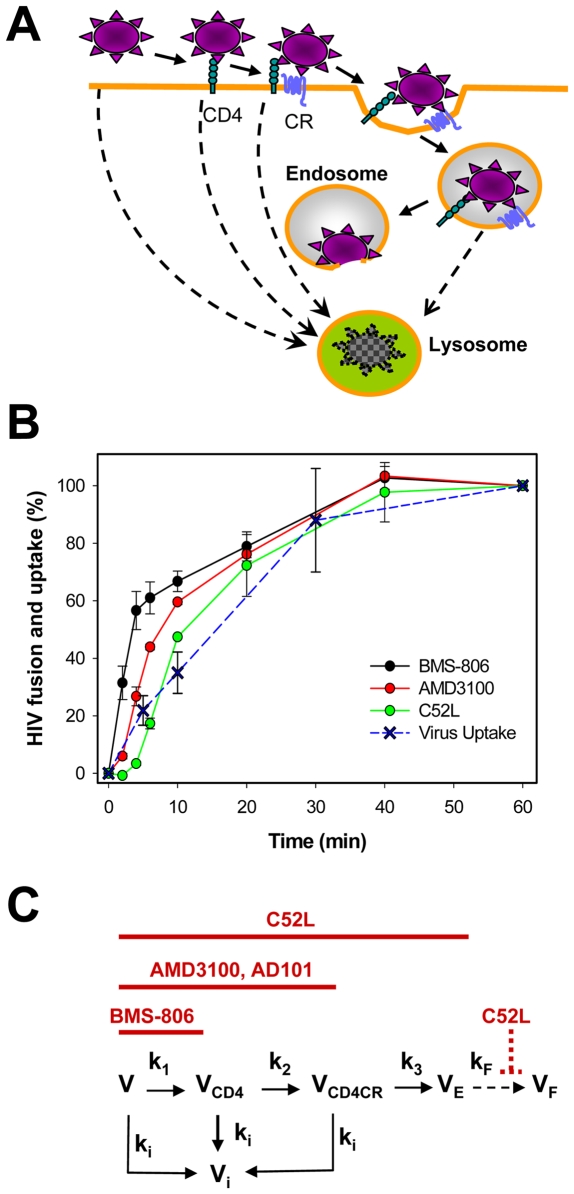
Dissection of early steps of HIV-1 fusion with target cells. (A) A schematic drawing showing HIV-1 entry via receptor-mediated endocytosis followed by fusion with endosomes. Pathways leading to virus degradation in lysosomes are shown by dashed lines. CR denotes coreceptor. (B) Kinetics of surface-accessible steps of HIV-1 fusion with TZM-bl cells. HXB2 pseudoviruses were pre-bound to cells in the cold and triggered to fuse by raising the temperature at time = 0. Inhibitors of CD4 binding (BMS-806), coreceptor binding (AMD3100) and the 6-helix bundle formation (C52L) were added at varied times of incubation to block the respective steps of fusion. The resulting extent of fusion as a function of incubation time was measured by a BlaM assay. The total virus uptake was measured by the fraction of pronase-resistant viral p24 (see Materials and Methods). Error bars are SEM. (C) A kinetic model of HIV fusion. The virus (V) progresses through CD4 and coreceptor (CR) binding steps and undergoes endocytosis (V_E_) that leads to fusion (V_F_). Stages of fusion sensitive to inhibitors, BMS-806, AMD3100 (CXCR4 binding), AD101 (CCR5 binding), and C52L are indicated by horizontal red lines. In this scheme, protection from C-peptides occurs via receptor-mediated endocytosis, but not through 6-helix bundle formation and fusion with the plasma membrane. Native viruses and those that engage CD4 and CD4/coreceptor are assumed to be inactivated with the rate constant k_i_, primarily due to a non-productive endocytosis.

The availability of inhibitors blocking distinct steps of HIV entry enabled monitoring the progression through sequential surface-accessible stages of fusion. The kinetics of CD4 binding were monitored by time-of-addition experiments using a small-molecule inhibitor BMS-806 [24],[25]. Since the binding to CD4 renders HIV-1 resistant to this compound, escape from BMS-806 indicates the progression of fusion beyond the receptor-dependent steps (Fig. 1B). The virtually complete inhibition of fusion by BMS-806 added at the beginning of incubation shows that cell-associated HIV-1 did not engage CD4 immediately following the virus pre-binding protocol (30 min at 4°C). The rates of CXCR4 or CCR5 binding were measured by adding small-molecule inhibitors AMD3100 and AD101, respectively.

The acquisition of resistance to C-peptides blocking the gp41 6HB formation has been traditionally interpreted as Env-mediated fusion [3],[4]. However, since HIV-1 fuses with endosomes of HeLa-derived target cells [15], escape from C-peptides must occur through virus uptake. The fact that HIV-1 escapes from coreceptor antagonists before escaping from C-peptides (Fig. 1B) implies that the fusion signal originates from viruses that engage both CD4 and coreceptors prior to undergoing endocytosis. Note that in HeLa-derived target cells the majority of viruses is internalized and degraded through CD4- and/or coreceptor-independent pathways [26]. It is unlikely that HIV-1 acquires resistance to these peptides by forming 6HBs prior to undergoing endocytosis because: (i) fusion with the plasma membrane does not progress beyond the lipid mixing stage [15]; and (ii) 6HB formation occurs only after opening of a fusion pore [13]. Here, we measured the rate of HIV-1 escape from C-peptides using a recombinant 52-residue peptide, C52L, derived from the gp41 C-HR domain [27]. In control experiments (data not shown) HIV-1 escape from the C52L peptide occurred at the same rate as escape from the better characterized 34-residue peptide, C34.

In this work, we will be concerned only with the pre-fusion steps occurring at the cell surface, which are key determinants of the sensitivity to C-peptides and antibodies targeting intermediate conformations of Env [9],[10],[18],[28],[29]. Once different HIV-1 isolates form ternary complexes with CD4 and coreceptors and undergo endocytosis, subsequent fusion events appear to occur with similar rates and efficiencies [15]. Thus, the fusion signal measured by the time-of-addition protocol should reflect the rate of cell surface-accessible steps of fusion.

### A model for HIV-1 fusion

We sought to determine the residence time of HIV-1 in key intermediate states and thus to evaluate the lifetime of gp41 pre-bundles that are accessible on the cell surface to C-peptides and neutralizing antibodies. To this end, we considered a minimal kinetic model (Fig. 1C) that describes the virus association with the cell surface followed by CD4 binding, coreceptor (CR) binding, and, finally, by productive endocytosis. We operationally define the following intermediate states the virus adopts sequentially along its entry pathway: (i) the state (V) of the membrane-associated virus which is sensitive to all three types of fusion inhibitors; (ii) the state (V_CD4_) resistant to an inhibitor blocking the HIV-CD4 binding, but sensitive to inhibitors of coreceptor binding and 6HB formation; (iii) the state V_CD4CR_ resistant to inhibitors of receptor and coreceptor binding, but still sensitive to inhibitors of 6HB formation; and (iv) the state V_E_ resistant to all three inhibitor types. The effective rate constants of transitions between the successive states V, V_CD4_, V_CD4CR_ and V_E_ are denoted by k_1_, k_2_, and k_3_. Our model can be readily modified to describe direct virus fusion with the plasma membrane by omitting the endosomal fusion step and treating V_E_ as the fusion state.

The model does not consider the reverse rates of CD4 and coreceptor binding reactions. Moreover, the model makes no assumptions about the stoichiometry of the receptor and coreceptor binding. If several Env glycoproteins are involved in HIV-1 entry, the virus must engage more than one pair of receptor and coreceptor molecules in order to undergo fusion. In that case, interactions with coreceptors at the virus-membrane contact may start before the recruitment of a requisite number of CD4 is completed. Then the V_CD4_ is a state where the receptor binding is finalized while the coreceptor recruitment is still incomplete, so that the transition to V_CD4CR_ consists in the recruitment of the missing coreceptors.

The model postulates that the viruses are subject to inactivation characterized by the inactivation rate constants. Whereas the general form of the model (see Fig. S4) accounts for different inactivation rates at the sequential steps of the HIV-1 progression along the fusion pathway, it is currently impossible to experimentally determine the individual inactivation rates. Thus, in order to evaluate the effective rate constants of fusion, we used a simplified version of the model that assumes equal inactivation rate constants, k_i_, for all stages of the fusion reaction. We also assume that the HIV-1 inactivation is primarily due to a non-productive endocytosis which is the predominant pathway of HIV-1 uptake by HeLa-derived cells [15],[26]. In other words, the total virus uptake is assumed to reflect the rate of virus inactivation. This non-productive pathway likely includes both CD4-independent and CD4-mediated virus uptake which, in the absence of coreceptor binding, does not lead to endosomal fusion.

The differential equations describing the virus evolution through each of the states of the kinetic scheme (Fig. 1A, C) are given in Appendix S1. The solution of these equations for the number of viruses V_E_ entering through productive endocytosis (leading to endosomal fusion) as a function of time is given by:
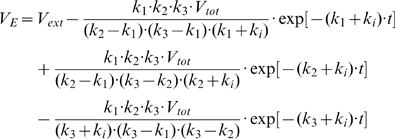
where 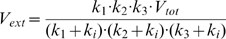


Unlike the previously proposed models of Env-mediated fusion [23],[30], this model accounts for the effective lag before fusion. This lag is given by 1/(k_1_·k_2_·k_3_·V_tot_), where V_tot_ is the total number of cell-bound fusion-competent viruses at time = 0. Note that V_tot_ cancels out upon normalizing data to the final extent of fusion (see also Fig. S1), so that the kinetics of fusion do not depend on the multiplicity of infection (MOI). Thus, normalization eliminates the need to determine and/or control the exact number of fusion-competent particles bound per cell. Our model describes a wide variety of cell-cell and virus-cell fusion data using four free parameters: k_1_, k_2_, k_3_ and k_i_. These effective rate constants can be defined through the measurements of the kinetics of CD4 binding (escape from BMS-806), coreceptor binding (escape from AD101 or AMD3100), the rate of productive endocytosis (escape from C52L), and the rate of virus inactivation through non-productive endocytosis (p24 uptake).

**Figure 2:**
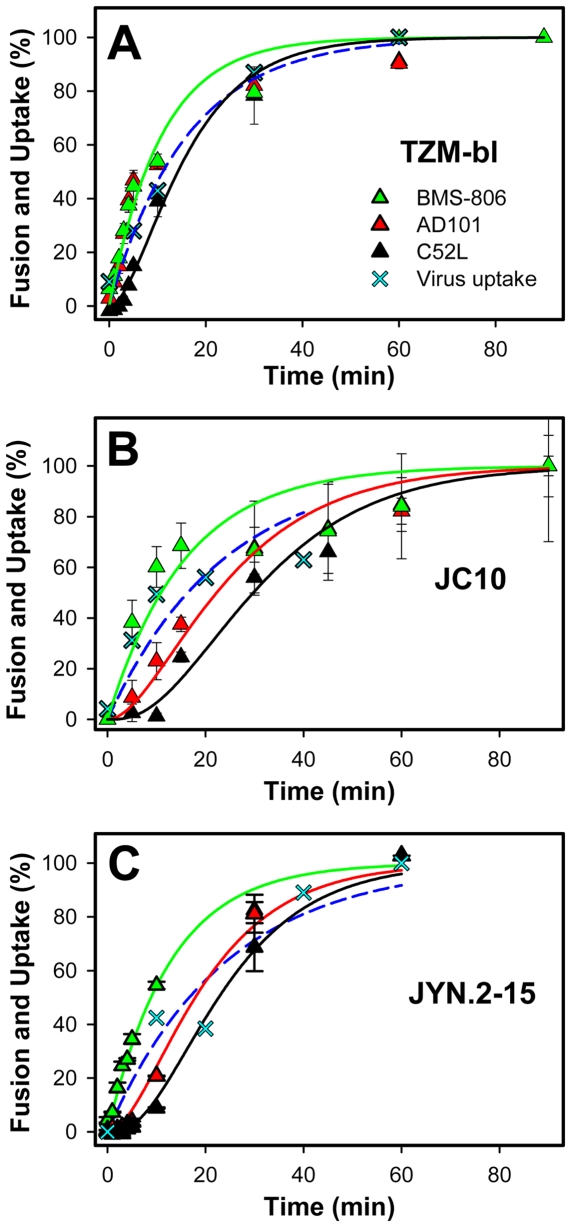
Kinetics of JRFL fusion with target cells expressing different levels of wild-type and mutant CCR5. (A) Fusion of JRFL pseudoviruses with TZM-bl cells was measured by adding inhibitors of distinct steps of this process (BMS-806, AD101 and C52L) at indicated time points, as described in the legend to Figure 1B. (B) JRFL fusion with HeLa-derived JC.10 cells expressing a low level of wild-type CCR5. (C) JRFL fusion with JYN.2-15 cells expressing a high level of mutant (Y14N) CCR5. Solid lines are the results of fitting the equations (10–12) of Appendix S1 to experimental data. The inactivation rate, k_i_, was determined by fitting a single-exponential function (dashed line) to the p24 uptake data (crosses). The k_1_ was obtained by curve fitting the expression (10) for the probability of V_CD4_ to the rate of escape from BMS-806 (after entering the k_i_ value and assigning arbitrary values to k_2_ and k_3_). The k_i_ and k_1_ were then used to obtain k_2_ by fitting the equation (11) for V_CD4CR_ to coreceptor binding data. Finally, these three rate constants were used to determine k_3_ through curve-fitting the equation (12) for the probability of V_E_ to the experimentally determined rate of virus escape from C52L. The lines are color-coded according to the symbols. Because the rates of JRFL escape from BMS-806 and AD101 were identical when TZM-bl cells were used as targets (panel A), only the CD4 binding curve (green line) is shown. Curve fitting was done using the SigmaPlot Regression Wizard (SYSTAT Software, Inc.).

The knowledge of the kinetics of virus inactivation is important because this process, along with the respective pre-fusion rate constants, determines the exit rates from V, V_CD4_ and V_CD4CR_. We thus obtained the k_i_ by fitting a single exponential function to the HIV p24 uptake data (Fig. 1B). A separate set of equations (equations (10–12) of Appendix S1) was derived to describe the kinetics of virus escape from CD4 and coreceptor binding inhibitors and from C-peptides added at varied times of virus-cell incubation. Through fitting of the solutions of these equations to the respective data sets, we were able to determine the remaining three rate constants k_1_, k_2_ and k_3_ for the surface-accessible steps of fusion (see the legend to Fig. 2 for details and Table 1).

**Table 1:** Effective rate constants of HIV-1 fusion and inactivation.

Virus	Cells	k_i_ (min^−1^)	k_1_ (min^−1^)	k_2_ (min^−1^)	k_3_ (min^−1^)
JRFL	TZM-bl	0.06±0.01*	0.03±0.01	>100	0.09±0.03
	JYN.2-15	0.04±0.02	0.04±0.01	0.07±0.02	0.17±0.05
	JC.10	0.04±0.04	0.02±0.01	0.05±0.02	0.10±0.04
HXB2	TZM-bl	0.05±0.01	0.09±0.01	0.15±0.02	0.25±0.04
BaL	TZM-bl	0.03±0.02	0.011±0.004	>100	>100
V3BaL	TZM-bl	0.05±0.01	0.09±0.01	0.48±0.1	0.13±0.04

*: Standard error of the curve fit.

### The lifetime of Env-CD4 complexes can be extended by lowering the density or the affinity of coreceptors to Env

We first examined the progression of the primary R5-tropic JRFL isolate through the surface-accessible fusion intermediates. Pseudoviruses were produced and the incorporation and proteolytic processing of JRFL Env was assessed by Western blotting (Fig. S2). Viruses were pre-bound to target cells in the cold, and their uptake and fusion were initiated by shifting to 37°C. Inhibitors of CD4 or CCR5 binding were added at indicated times of incubation to obtain the kinetics of HIV-1 escape from these inhibitors. As discussed above, HIV-1 acquires resistance to the inhibitors of 6HB formation through receptor-mediated endocytosis as opposed to fusion at the cell surface. Productive endocytosis of this virus was thus measured by adding a high concentration of the recombinant C52L peptide at varied time points. In parallel experiments, the total virus uptake was measured by the intracellular accumulation of the HIV-1 p24. These measurements allowed us to determine the fusion and inactivation rate constants for JRFL entry into different target cells.

When HeLa-derived TZM-bl cells expressing high levels of CD4 and CCR5 [31] were used as targets, JRFL engaged a requisite number of CD4 and coreceptors with the virtually identical kinetics (Fig. 2A). Thus, under these conditions, the effective rate of CCR5 binding was too fast to be resolved. We therefore sought to slow down the formation of ternary complexes by reducing the density of CCR5 on the cell surface or by diminishing its affinity to Env. A ∼20-fold reduction of the average number of CCR5 molecules per cell did not noticeably affect the rate of coreceptor engagement (data not shown). However, the kinetics of CCR5 binding and the final extent of fusion were markedly diminished for JC.10 cells [31] expressing a ∼100-fold lower number of CCR5 compared to TZM-bl cells (Fig. 2B). We were thus able to kinetically resolve the CCR5 binding step and measure its effective rate constant, k_2_ (Table 1).

Next, we examined the impact of the coreceptor binding affinity on the rate of the ternary complex formation. JRFL pseudoviruses were bound and fused with JYN.2-15 cells [32] expressing high levels of wild-type CD4 and the Y14N CCR5 mutant similar to those present on TZM-bl cells. JRFL fusion with these cells was less efficient and was highly sensitive to inhibition by AD101 (Table 2), in agreement with the diminished affinity to gp120 caused by the loss of the critical Tyr14 residue at the CCR5 N-terminus. As expected, the Y14N mutation reduced the kinetics of virus escape from AD101, whereas the rate of CD4 binding remained unchanged (Fig. 2C and Table 1). Together, these results demonstrate that the lifetime of Env-CD4 complexes on the cell surface can be manipulated by changing the density of coreceptors or their binding affinity to gp120.

**Table 2:** HIV-1 sensitivity to inhibition by AD101 and C34.

Viruses	Cells	IC_50_ AD101 (nM)	IC_50_ C34 (nM)
JRFL	TZM-bl	390±87*	15±3 (8±1***)
	JC10	ND**	7±1
	JYN.2-15	<0.1	8±2
HXB2	TZM-bl		1.6±0.2
	JYN.2-15		5.1±0.2
BaL	TZM-bl	170±33	8±1
V3BaL	TZM-bl	710±207	2.3±0.3
	JYN.2-15	ND	1.7±0.2

*: Standard error of the curve fit.

**: ND, not determined.

***: IC_50_ for the C34_JRFL_ peptide.

### Increased coreceptor binding affinity in the context of X4 Env accelerates the coreceptor engagement but not HIV-1 endocytosis

**Figure 3:**
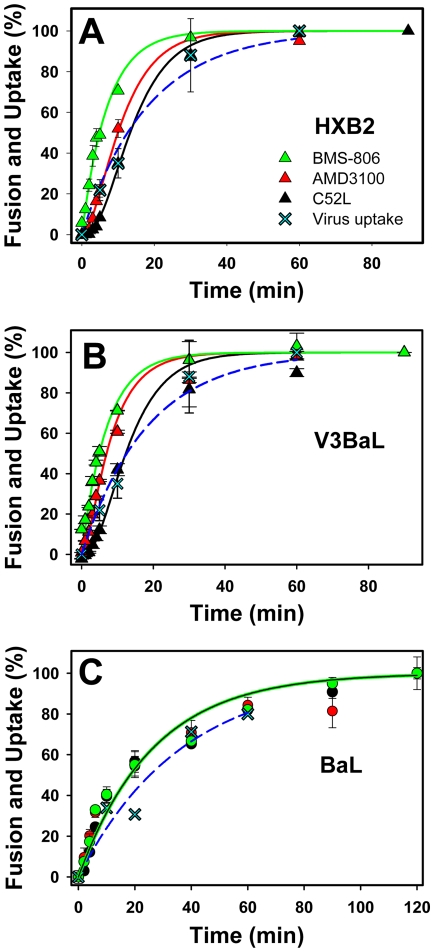
Progression of the HXB2, V3BaL and BaL through surface-accessible stages of fusion. (A) The kinetics of HXB2 binding to CD4 and CXCR4 was determined by the time-of-addition experiments using BMS-806 and AMD3100 (see also Fig. 1B), and the rate of productive endocytosis was measured by adding C52L. (B, C) The rates of CD4 and CCR5 engagement by V3BaL (B) and BaL (C) were determined by adding BMS-806 and AD101, respectively. The kinetics of receptor-mediated virus endocytosis was measured by escape from C52L. In all panels, the bulk HIV-1 uptake was determined by accumulation of the intracellular p24 (cyan crosses) and fitted with a single-exponential function (dashed lines). The solid lines were obtained by curve fitting the model equations to experimental points, as described in the text and in the legend to Figure 2. Since all early steps of BaL fusion occurred virtually simultaneously, only one theoretical curve is shown in panel C.

Next, we compared the kinetics of surface-accessible steps of fusion induced by JRFL Env and by the laboratory adapted HXB2 Env. These glycoproteins differ in many aspects, including the coreceptor tropism and sensitivity to neutralizing antibodies and C-peptides [18],[33],[34]. HXB2 pseudoviruses engaged CD4 faster than JRFL (Figs. 2A and 3A), in agreement with the enhanced receptor binding affinity associated with the HIV-1 adaptation to growth in culture [5],[35],[36]. By contrast, HXB2-CD4 complexes became protected from the coreceptor binding inhibitor at a much slower rate than JRFL. The different kinetics of coreceptor binding are consistent with the vastly different coreceptor binding affinities of laboratory adapted X4 and primary R5 Env glycoproteins [37]–[39]. The ∼10-fold higher CCR5 expression on TZM-bl cells compared to the CXCR4 expression [31],[40] did not seem to be responsible for the more rapid CCR5 engagement, since the rate of this coreceptor binding was not noticeably affected by the 10-fold reduction in its expression level (data not shown). Interestingly, HXB2-CD4-coreceptor complexes were internalized at a ∼3-fold faster rate compared to the ternary JRFL complexes (k_3_ constant, Table 1), whereas the rates of bulk endocytosis leading to virus degradation were close for these viruses (Figs. 2A and 3A, crosses). As a result of the above compensatory variations in the rate constants of pre-fusion steps, the overall kinetics of JRFL and HXB2 escape from C52L were close. This result is in contrast with the faster kinetics of cell-cell fusion induced by JRFL compared to HXB2 Env [18].

We next assessed the effect of coreceptor binding affinity in the context of X4 Env. Toward this goal, we used a chimeric HXB2 Env in which the V3 loop was substituted with that of the R5-tropic BaL isolate [41]. This chimera, hereafter referred to as V3BaL, exclusively utilizes CCR5 for fusion and appears to bind CCR5 with high affinity [21],[41]. This notion is consistent with our data showing that V3BaL fuses with target cells expressing the low-affinity CCR5 mutants (Fig. S3A) and with cells expressing low density of CCR5 (data not shown). Moreover, V3BaL was even somewhat more resistant to AD101 than BaL and JRFL (Table 2).

After verifying that HXB2, V3BaL and BaL Env were proteolytically processed and incorporated into pseudoviruses at similar levels (Fig. S2), we compared the kinetics of fusion mediated by these glycoproteins. As expected, the rates of CD4 binding by V3BaL and HXB2 were indistinguishable (Fig. 3A, B and Table 1), whereas the V3BaL-CCR5 binding was ∼3-fold faster than HXB2-CXCR4 binding. This result is consistent with the high affinity of the chimera to CCR5 and with the higher expression of CCR5 on TZM-bl cells compared to endogenous expression of CXCR4. In spite of the faster rate of ternary complex formation, the subsequent internalization of V3BaL-CD4-coreceptor complexes was ∼2-fold slower than that of HXB2 (Table 1). These opposite trends in the kinetics of the HXB2 and V3BaL pre-fusion steps resulted in similar overall rates of escape from C52L.

To better understand the differences in the progression of HXB2 and V3BaL through surface-accessible steps of fusion, we examined the fusion of viruses pseudotyped with wild-type BaL Env. BaL exhibited markedly different kinetics of fusion compared to HXB2 and V3BaL (Fig. 3). First, BaL engaged CD4 ∼3-fold slower than JRFL and almost 9-fold slower than HXB2 (Table 1). By contrast, BaL escaped from AD101 and from C52L at rates that were indistinguishable from the rate of CD4 binding. The unusually quick BaL protection from C52L prompted us to examine whether this effect was due to the direct fusion with the plasma membrane as opposed to productive endocytosis demonstrated for JRFL and HXB2 viruses [15]. We therefore compared the rates of virus escape from C52L and from the temperature block applied at varied times of BaL incubation with TZM-bl cells (see [15] and Fig. S3B). A marked delay between the BaL escape from the temperature block compared to its escape from the peptide inhibitor strongly implies that this virus also fuses with endosomes. Collectively, these data show that BaL engages CD4 slowly but then completes the CCR5 binding and enters endocytic compartments at an unusually high rate.

### The lifetime of gp41 pre-bundles can be obtained from the rates of coreceptor engagement and productive endocytosis

**Figure 4:**
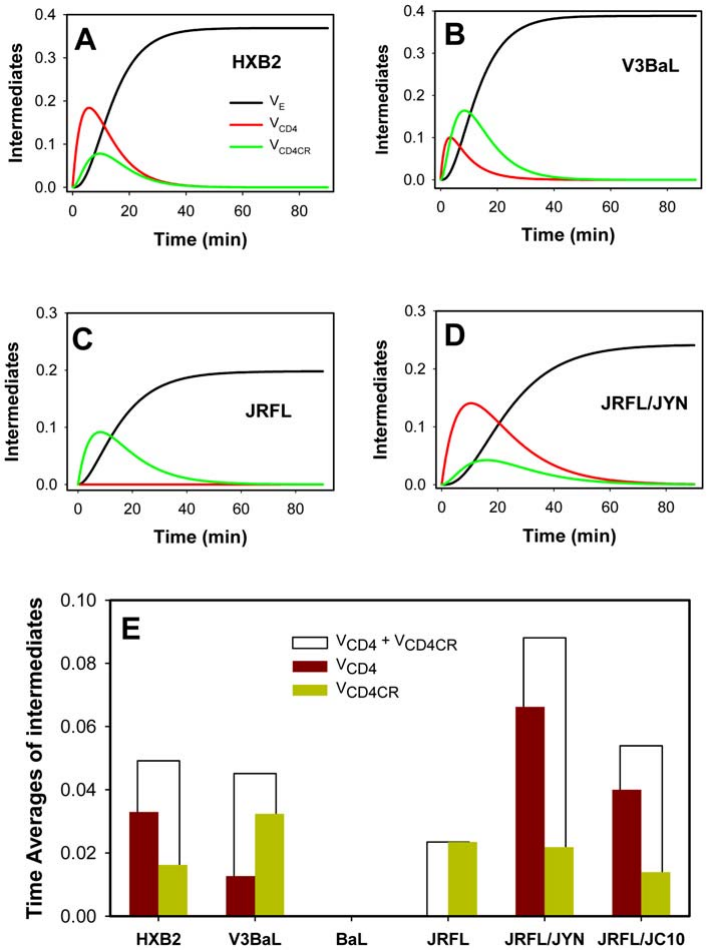
Kinetics of HIV-1 progression through intermediate states and the residence times in these states. (A–D) The probabilities of finding the virus in CD4-bound (V_CD4_) and coreceptor-bound (V_CD4CR_) states and of undergoing receptor-mediated endocytosis (V_E_) as a function of time were obtained by entering the respective rate constants (Table 1) into the equations (6–8). (E) Time averages for HIV-1 in the CD4-bound and coreceptor-bound states were determined from equations (13) and (14), using the respective rate constants. The total time averages of HIV-1 in intermediate stages prior to undergoing endocytosis (the sum of V_CD4_ and V_CD4CR_) are shown by open bars.

Having determined the effective rate constants of progression through the CD4 and coreceptor binding steps for different Env glycoproteins and target cells (Table 1), we used the equations (6–8) of Appendix S1 to calculate the probability of finding the virus in V_CD4_, V_CD4CR_ and V_E_ states as a function of time (Fig. 4A–D). We then determined the time averages of HIV-1 in these states by integrating the equations (6) and (7) from time = 0 to the end of virus-cell incubation and dividing over this time interval (the resulting equations (13) and (14) are given in Appendix S1). The time averages of different HIV-1 in V_CD4_ and V_CD4CR_ and the combined residence times in both states are shown in Fig. 4E.

The gp41 pre-bundles are formed upon the Env binding to CD4 or both CD4 and coreceptors [2]–[4] and are cleared from the cell surface by endocytosis [15]. Thus, the ability to determine the time spent in V_CD4_ and V_CD4CR_ states provided an opportunity to estimate the lifetimes of pre-bundles on the cell surface. The shortest apparent exposure of pre-bundle intermediates was observed for BaL, whereas the longest combined time in V_CD4_ and V_CD4CR_ was observed upon JRFL fusion with cells expressing the low-affinity CCR5 mutant (Fig. 4E). Since C-peptides target the gp41 coiled coils, we asked whether their inhibitory potency correlated with the lifetime of these intermediates on the cell surface. It has been proposed that the longevity of pre-bundles determines the HIV-1 sensitivity to C-peptides [9]. However, this notion was based on correlation between the peptide's potency and the overall kinetics of HIV Env-mediated cell-cell fusion and not on measurements of the actual lifetime of gp41 coiled coils in the context of virus entry. In order to rationalize the kinetic determinants of HIV-1 sensitivity to C-peptides, we sought to compare the time spent in pre-bundle intermediates with the inhibitory potency of these peptides.

### HIV-1 resistance to C-peptides does not correlate with the rate of productive endocytosis

**Figure 5:**
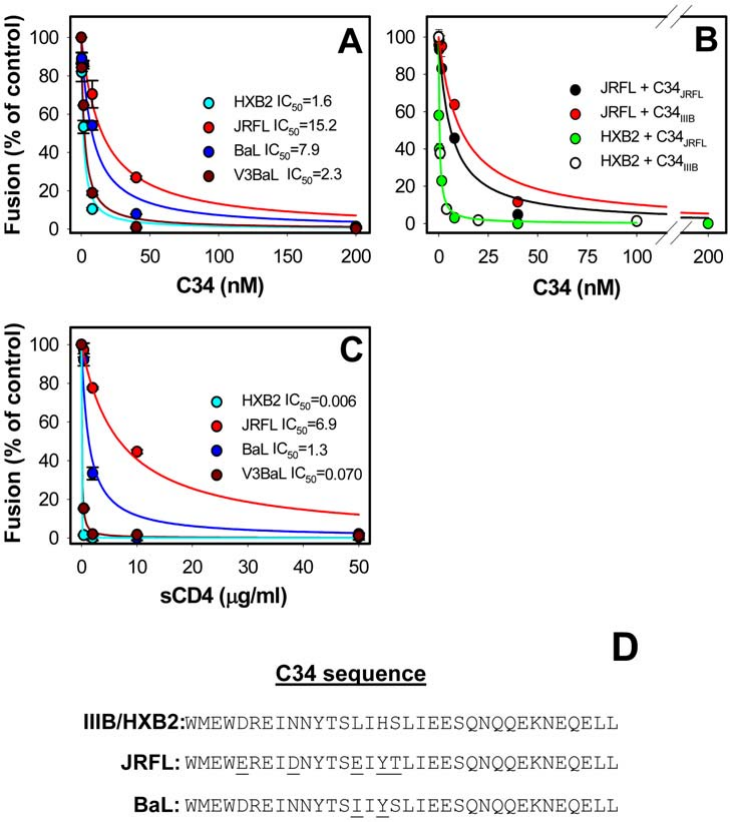
HIV-1 inhibition by the C34 peptide and inactivation by soluble CD4. (A) Viruses were pre-bound to TZM-bl cells and allowed to fuse for 90 min in the presence of varied concentrations of C34 derived from the HIV-1 IIIB strain. The solid lines were obtained by curve fitting experimental data using the following equation: Fusion = 1/(1+[C34]/IC_50_), where [C34] is the C34 concentration (see Table 2 for the IC_50_ values). (B) Viruses were fused with TZM-bl cells in the presence of different concentrations of C34 derived from either IIIB or JRFL gp41 (denoted C34_IIIB_ and C34_JRFL_, respectively). (C) HIV-1 inactivation by different doses of soluble CD4 was carried out as described in Materials and Methods. (D) Amino acid sequences of the C34 peptide derived from the IIIB/HXB2, JRFL and BaL gp41. The error bars are SEM of at least three independent measurements.

To determine the susceptibility of different HIV-1 Env to inhibition by C-peptides, we measured virus-cell fusion in the presence of the well-characterized C34 peptide [19]. JRFL exhibited the highest resistance to this peptide while BaL was somewhat more susceptible to inhibition (Fig. 5A, Table 2). By comparison, HXB2 and V3BaL were much more potently inhibited by C34 with the chimera being only marginally more resistant than the wild-type HXB2. So, in the context of HXB2 Env and under our experimental conditions, the coreceptor tropism and the coreceptor binding affinity had a modest effect on the virus' resistance to C34. Similar results were obtained with the C52L peptide (data not shown).

**Figure 6:**
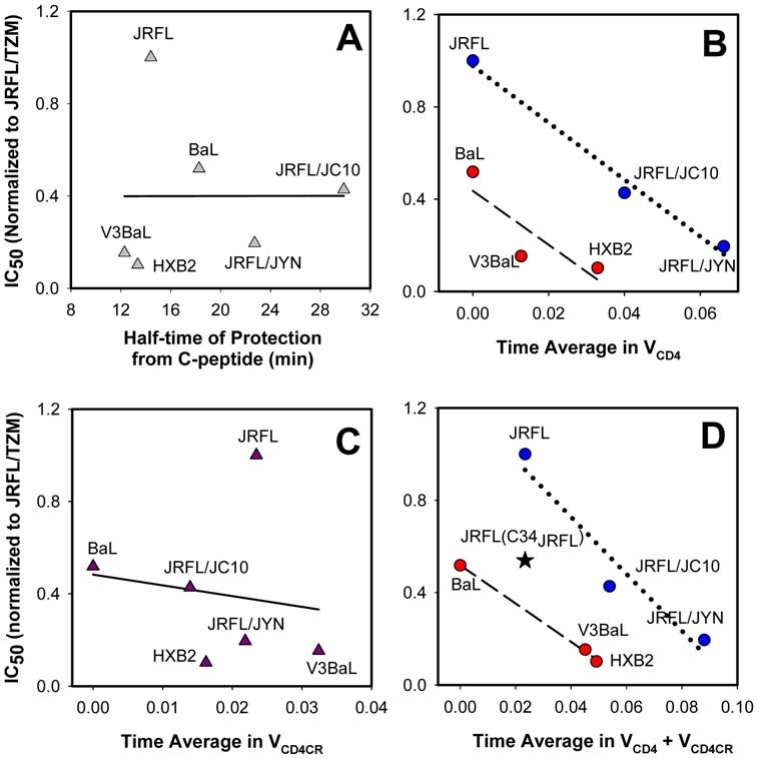
Correlation between the potency of C34 and the time average of HIV-1 in intermediate states. (A) Relationship between the C34 IC_50_ (normalized to that of JRFL on TZM-bl cells) and the kinetics of escape from a high concentration of C52L parameterized as the time at which half of the viruses acquire resistance to this peptide. Solid line is a linear regression. (B) The C34 IC_50_ was normalized to that for the JRFL fusion with TZM-bl cells and plotted vs. the time average in the V_CD4_ state. Separate linear regressions are shown for JRFL fusion with different target cells (dotted line, r^2^ = 0.99) and for HXB2-BaL-V3BaL constructs fusing with TZM-bl cells (dashed line, r^2^ = 0.73). (C) Lack of correlation between the inhibitory activity of C34 and the lifetime of the V_CD4CR_ state. (D) Correlation between the time averages in both intermediate stages (V_CD4_ plus V_CD4CR_) and the potency of C34. Separate linear regressions are shown for BaL, HXB2 and V3BaL (dashed line, r^2^ = 1.00) and for JRFL fusion with different target cells (dotted line, r^2^ = 0.93). The IC_50_ for the C34_JRFL_ against JRFL (star) is shown for comparison. Note that the more potent C34_JRFL_ peptide inhibited JRFL almost as efficiently as the C34_IIIB_ inhibited BaL (Table 2).

The 10-fold difference between the IC_50_ for JRFL and HXB2 by C34 is in stark contrast with the comparable rates of their escape from high doses of C-peptides due to productive endocytosis (Figs. 2A and 3A). Plotting the IC_50_ values against the half-times of HIV-1 escape from C52L, as determined from the measurements shown in Figs. 2 and 3, confirmed that the C34 potency did not correlate with the rate of virus protection from this inhibitor (Fig. 6A). In contrast to these results, the kinetics of cell-cell fusion appears to inversely correlate with the potency of C-peptides [9]. These seemingly discrepant findings likely stem from the fact that HIV-1 escape from inhibitory peptides does not reflect virus-cell fusion, but rather corresponds to productive endocytosis.

Another possibility is that the C34 peptide used in our experiments had different affinity to the gp41 N-HR domains of HIV-1 isolates examined in this work. The conventional C34 peptide is derived from the gp41 C-HR of the IIIB clone [17],[19], which is identical to the respective domains of HXB2 gp41 but not of JRFL gp41 (Fig. 5D). Since the C34 sequence has been shown to affect its potency against HIV and SIV isolates [17], we asked if the JRFL-derived C34 (designated C34_JRFL_) is a more potent inhibitor of fusion than the C34_IIIB_. Both C34_IIIB_ and C34_JRFL_ potently inhibited HXB2 fusion at comparable concentrations (Fig. 5B). Even though JRFL was approximately 2-fold more sensitive to its own C34 than to C34_IIIB_, still higher doses of the former peptide were required to block JRFL fusion compared to HXB2 fusion. Thus, neither the kinetics of virus escape from C-peptides nor the sequences of C34 can fully account for the greater resistance of JRFL to this inhibitor. The above results highlight the importance of measuring the actual lifetime of gp41 pre-bundles (Fig. 4) in order to better rationalize the kinetic factors controlling the HIV-1 resistance to C-peptides.

### Kinetic determinants of HIV-1 resistance to C-peptides

We asked whether the time averages of V_CD4_ and V_CD4CR_ states are predictive of the virus' sensitivity to C-peptides. When the potency of C34 was plotted against the time spent in V_CD4_ (Fig. 6B), the points clearly fell into two groups – JRFL fusion with different target cells and HXB2/BaL/V3BaL fusion with TZM-bl cells. Within these two groups, the IC_50_ correlated well with the time average in V_CD4_. Thus, in the context of JRFL Env or HXB2/BaL Env, the diminished rate of coreceptor engagement and thus the increased lifetime of early gp41 intermediates were associated with enhanced sensitivity to C34. In contrast, we saw no apparent correlation between the lifetime of V_CD4CR_ and the potency of this peptide (Fig. 6C). This was surprising because the gp41 coiled coils should be better exposed to the peptide at V_CD4CR_ compared to V_CD4_ [2].

Since the HXB2 (and likely V3BaL) coiled coils are exposed as early as upon CD4 binding [2]–[4] and should thus persist throughout the surface-accessible steps of fusion, a more meaningful parameter for characterizing the window of opportunity for the C34 binding is the total time spent in V_CD4_ and V_CD4CR_ states. This parameter should also adequately describe the coiled coil exposure on JRFL and BaL gp41. Even though the JRFL coiled coils are optimally exposed only after engaging both CD4 and CCR5 [2], the total time in V_CD4_ and V_CD4CR_ states is dominated by the latter intermediate (Fig. 4C and E). We found that the combined residence time in these states inversely correlated with the IC_50_ for C34 (Fig. 6D). As in the Fig. 6B, this correlation was more apparent when fusion of the V3 loop-swapped constructs (BaL, HXB2 and V3BaL) and JRFL fusion with different cells were considered separately. These data suggest that, in the context of the same Env backbone, the total time spent in V_CD4_ and V_CD4CR_ is a good predictor of the C34's potency.

### The extent of Env inactivation by soluble CD4 correlates with its sensitivity to C-peptides

The lifetime of the gp41 coiled coils did not fully account for the differences in the inhibitory potency of C34. Indeed, the quick progression of BaL through the surface-accessible pre-fusion steps suggests that the gp41 coiled coils are very briefly exposed prior to entering into endosomal compartments. However, whereas BaL was much more resistant to C34 than HXB2 (Table 2), it was more susceptible to this inhibitor than JRFL, which spent much longer time in pre-bundle intermediates than BaL. Thus, virus strain-specific factors appear to contribute to the baseline sensitivity of HIV-1 to C-peptides. In addition, the modest effect of the HXB2 V3 loop substitution on the potency of C34 indicates that critical determinants of the virus' resistance reside outside the V3 loop. Envelope glycoproteins of laboratory adapted strains are generally less stable and tend to inactivate upon binding to CD4 [42]–[44]. It is thus possible that the degree to which different Env glycoproteins refold in response to the CD4 binding could determine the sensitivity to C-peptides [17]. Recent study implies that this inactivation occurs via CD4-induced conformational changes in Env and not due to the gp120 shedding [45]. This work also demonstrates the strain-dependent differences in the exposure of the gp41 coiled coils caused by sCD4 binding.

To assess the extent of irreversible Env refolding in response to receptor binding, we measured to degree of virus inactivation after pre-treatment with sCD4. Viruses were pre-incubated with varied concentrations of sCD4, and the remaining fusion activity was determined after an additional 90 min-incubation with TZM-bl cells. These experiments revealed that JRFL was the most resistant to sCD4, BaL showed an intermediate sensitivity, whereas V3BaL and HXB2 were both strongly inactivated under these conditions (Fig. 5C). The similar effect of sCD4 on HXB2 and V3BaL viruses was as expected for Env glycoproteins sharing the same backbone.

Notably, the extents of sCD4-induced inactivation and inhibition by C-peptides ranked similarly (Fig. 5A and C). This result is consistent with the notion that the stability of Env-CD4 complexes determines the extent of exposure of the gp41 coiled coil. In other words, for the same time spent in a pre-bundle conformation, the sensitivity to C-peptides appears to depend on the extent of conformational changes in Env occurring in response to the receptor binding. This model is supported by the different efficacies of C34 against HXB2 fusing with TZM-bl and JRFL fusing with JC.10 cells in spite of the similar lifetimes of gp41 pre-bundles (Fig. 6D). Together, our results reveal a complex interplay between the Env stability (viral determinants) and the rate of progression through surface-accessible intermediates (viral and cellular determinants) in defining the HIV-1 resistance to C-peptides.

## Discussion

In the present work, we characterized the HIV-1 progression through surface-accessible steps of entry prior to virus uptake and fusion with endosomes. Through measuring the rates of escape from membrane-impermeant inhibitors blocking distinct pre-fusion steps, we were able to determine the residence times in intermediate states in which HIV-1 recruited a requisite number of CD4 or both CD4 and coreceptors. This analysis revealed the viral strain-dependent and target cell-dependent differences in the average times the HIV-1 spent in distinct intermediates on the cell surface. The knowledge of the HIV-1 progression through the pre-fusion steps permitted the rationalization of the inhibitory potency of C-peptides targeting the gp41 coiled coils. We found that, unlike the Env-mediated cell-cell fusion, the time-course of HIV-1 escape from inhibitors of 6HB formation poorly correlated with the resistance to C34 (Fig. 6A). This could be due to the different rates of productive virus endocytosis that, in addition to the kinetics of CD4 and coreceptor engagement, control the time of exposure of the gp41 coiled coils on the cell surface.

Quantitative analysis of the HIV-1 fusion kinetics revealed correlation between the total residence time in pre-bundle conformations and the potency of C34. Interestingly, the lifetime of the V_CD4_, but not of V_CD4CR_, was predictive of the virus' sensitivity to this inhibitor. This result was unexpected because the gp41 coiled coils should be better exposed to C-peptides at the latter state [2]. The previously proposed model [9] posits that the C-peptide binding to the complementary coiled coil domain is slow and thus occurs optimally upon prolonged exposure of these domains. However, our data suggest that the peptides can effectively bind to relatively short-lived gp41 intermediates (Fig. 6B). The observation that C-peptides cause closure of nascent fusion pores shortly after their addition to fusing cells [13] also supports the notion that the peptide binding occurs on a shorter time scale than the average lifetime of a pre-bundle intermediate (several minutes). We surmise that the quick peptide binding to the stably exposed coiled coils can account for the lack of correlation between the lifetime of ternary complexes and the potency of C34 (Fig. 6C). On the other hand, the enhanced potency of C34 associated with the longer-lived V_CD4_ indicates that the coiled coil exposure at this point is not completed.

Our findings are in agreement with the previous reports that multiple factors control the HIV-1 resistance to C-peptides [17],[20]. First, the baseline sensitivity of diverse Env glycoproteins appears to be determined by their propensity to undergo irreversible conformational changes upon engaging CD4. The exceptionally high resistance of JRFL to C-peptides in spite of the long time spent in CD4/coreceptor-bound state could be due in part to a restricted exposure of its coiled coils [2]. Second, the C-peptide's primary sequence, which affects their binding affinity to the gp41 coiled coil, appears to modulate their inhibitory potency. Third, our results show that kinetic factors determine the longevity of gp41 pre-bundles and thus control the potency of C-peptides. The window of opportunity for the C-peptide binding depends on cellular factors, such as the density of coreceptors and their affinity to Env, as well as on the rate of receptor-mediated virus endocytosis. We surmise that the faster rate of productive endocytosis in certain cell types may diminish the gp41 pre-bundle exposure and protect the virus from C-peptides and antibodies targeting Env intermediates.

Clearly, a full quantitative description of HIV-1 fusion awaits the determination of the fusion stoichiometry, as well as the identification of intracellular steps and factors involved in virus entry. However, experimental strategies developed in this work provide a means to evaluate the kinetics of surface-accessible steps of HIV-1 fusion. Our data show that the rate of progression through pre-fusion steps is a critical determinant of the virus' susceptibility to C-peptides and likely to neutralizing antibodies targeting CD4-induced epitopes. Further studies involving a larger set of primary and laboratory adapted HIV strains are needed to substantiate the conclusions of this work and to define the viral and cellular determinants of resistance to peptide inhibitors.

## Materials and Methods

### Cells and reagents

HeLa-derived JC.10 [31], JYN.2-15, and JGR.H11 [32] cells were a gift from Dr. D. Kabat (OHSU, OR). TZM-bl cells were obtained from NIH AIDS Research & Reference Reagent Program and grown in Dulbecco modified Eagle medium (DMEM, Invitrogen, Carlsbad, CA) supplemented with 10% fetal bovine serum (FBS, HyClone Laboratories, Logan, UT) and penicillin-streptomycin (Invitrogen). 293T/17 cells (ATCC, Manassas, VA) were grown in DMEM/10% FBS, 0.5 mg/ml Geneticin (Invitrogen), and penicillin-streptomycin. The pCAGGS plasmids encoding JRFL or HXB2 Env [46] were provided by Dr. J. Binley (Torrey Pines Institute, CA). The pCAGGS plasmids encoding HXB2-BaL chimera Env in V3 loop, V3BaL, were constructed by replacing of Env coding sequence of pSV7D HXB BaL, a gift from Dr. R. Doms (University of Pennsylvania). HIV-1 BaL.01 [47] Env expression vector was obtained from NIH AIDS Research & Reference Reagent Program. The HIV-1 based packaging vector pR8ΔEnv lacking the *env* gene was from Dr. D. Trono (University of Geneva, Switzerland). Soluble CD4 was purchased from Progenics (Tarrytown, NY). The C52L recombinant peptide [27] was a gift from Dr. Min Lu (Cornell University). BMS-806 [24],[25] was synthesized by ChemPacific Corp. (Baltimore, MD), AMD3100 [48] and pronase were purchased from Sigma (St. Louis, MO), and AD101 [49] was a gift from Dr. J. Strizki (Schering Plough, Kenilworth, NJ). The C34_IIIB_ peptide was synthesized by Dr. L-X. Wang (IHV, University of Maryland) and the JRFL gp41-derived C34 peptide (Ac-WMEWEREIDNYTSEIYTLIEESQNQQEKNEQELL-NH_2_) was kindly provided by Dr. W. Lu (IHV, University of Maryland). The purity of these peptides was >98%, as determined by HPLC.

### Virus preparation

Pseudoviruses containing the β-lactamase-Vpr (BlaM-Vpr) were produced as described in [15]. Briefly, 293T/17 cells on a 60 mm dish were transfected by Ca-phosphate protocol with 10 µg pR8ΔEnv, 5 µg pMM310 vector expressing BlaM-Vpr [50], 4 µg pcRev [51], and 15 µg pCAGGS encoding JRFL, HXB2, or V3BaL Env, or pHIV1 BaL.01 (expressing BaL Env). The infectious titer was determined by a β-Gal assay in TZM-bl cells [52].

### Virus-cell fusion assay

Measurements of HIV-1 fusion with target cells were carried out essentially as described previously [15]. Briefly, viruses bearing the BlaM-Vpr chimera were bound to TZM-bl cells by centrifugation at 2095×g, 4°C for 30 min. After washing off unbound viruses, cells were incubated at 37°C for 90–120 min. At indicated times of incubation, the fusion reaction was stopped by adding specific inhibitors of CD4 binding (10 µM of BMS-806), coreceptor binding (5 µM of AMD3100 or 7 µM of AD101), or inhibitors of 6-helix bundle formation (1 µM of C52L). The concentration of a given entry inhibitor used in these time-of-addition experiments exceeded the fully inhibitory concentration (determined in separate experiments) by at least 3-fold. Samples were then loaded with the BlaM substrate CCF2-AM (GeneBLAzer *in vivo* detection kit, Invitrogen) on ice and incubated at 20°C for 12 hr. The resulting fluorescence signals at 460 nm (blue) and 528 nm (green) were measured using the Synergy HT plate reader (Bio-Tek Instr., Germany). The dose-response dependence for HIV fusion in the presence of inhibitors was obtained by pre-binding viruses to cells, as above, and incubating at 37°C for 90 min with various concentrations of C34 prior loading cells with the BlaM substrate.

### Virus internalization assay

Cells (3·10^4^ cells/well in 96-well plates) were grown overnight in regular medium. Virions were added to cells (MOI 0.7) and centrifuged as described above. After washing to remove unbound viruses, cells were incubated at 37°C for varied times in the presence of 1 µM of C52L to prevent fusion. At defined time points, virus uptake was stopped and the external virus was stripped off by treatment with 2 mg/ml pronase on ice for 10 min. After washing with DMEM/10% serum, cells were lysed and the amount of p24 in cell lysate was determined using a p24 ELISA Kit (PerkinElmer Life Sciences Inc, Boston, MA).

### Virus inactivation by soluble CD4

Twenty µl aliquots of 10-fold concentrated viral preparations were pre-incubated for 30 min at 37°C with different concentrations of sCD4. Following the incubation, the mixture was diluted 10-fold with the growth medium and added to TZM-bl cells (final MOI = 0.8 in the absence of sCD4). Virus binding to cells was augmented by centrifugation at 4°C, as described above. Cells were washed to remove unbound viruses, and fusion was initiated by shifting to 37°C for 90 min, after which time the process was stopped by reducing the temperature. The BlaM signal was normalized to that obtained for mock-treated viruses.

## Supporting Information

Figure S1 The normalized kinetics of fusion is independent of the number of cell-bound viruses. JRFL viruses were added to TZM-bl cells at the multiplicity of infection (MOI) 1 or 0.2. After pre-binding the viruses at 4°C, cells were washed and shifted to 37°C to induce fusion. (A) The kinetics of BlaM signal for the two different MOI is shown. (B) The kinetics of fusion after normalizing the data to the last time point. Even though the overall BlaM signal is proportional to the MOI, the rate of fusion is independent of the virus input.
(0.37 MB TIF)

Figure S2 Processing and incorporation of various HIV-1 Env glycoproteins into pseudoviruses assessed by Western blotting. Viral lysates were prepared with RIPA lysis buffer (0.05 M TrisCl, 0.15 M NaCl, 1% Triton X-100, 0.1% sodium dodecyl sulfate (SDS) and 1% sodium deoxycholate) that contained the Complete protease inhibitor (Roche Applied Science, Mannheim, Germany). The samples were separated on a 10% SDS-PAGE gel and transferred to PVDF membrane (Millipore, Billerica, MA). The viral proteins were then detected with anti-β-lactamase monoclonal antibody (QED Bioscience Inc, San Diego, CA), anti-p24 sera (from AIDS Research and Reference Reagent Program), and anti-gp120 polyclonal antibody (Fitzgerald, Concord, MA). The blot was treated with biotinylated anti-mouse or rabbit immunoglobulin (GE Healthcare Bio-Sciences AB, Uppsala, Sweden), or anti-goat immunoglobulin (Sigma) as the secondary antibody and then incubated with streptavidin-horseradish peroxidase conjugate (GE Healthcare Bio-Sciences). Chemiluminescence from the protein bands was visualized on the Fluor Chem SP (Alpha Innotech, San Leandro, CA) using the Lumi-Light^plus^ (Roche) substrate.
(1.04 MB TIF)

Figure S3 Fusion with target cells expressing mutant CCR5 and escape from C52L and the temperature block. (A) The efficacy of JRFL, HXB2, BaL and V3BaL fusion with HeLa cells expressing similar levels of CD4, CXCR4 and wild-type (TZM-bl) or mutant (JYN.2-15 and JGR.H11) CCR5. In these experiments, the viral input was normalized by inoculating the cells with the same amount of virus (an equivalent of MOI 0.7 for TZM-bl cells). Note that BaL failed to fuse with cells expressing the Y14N CCR5 and fused inefficiently with cells expressing the G163R mutant. The ability of V3BaL to utilize the Y14N CCR5 mutant is consistent with the less pronounced dependency of X4-tropic viruses on the N-terminal segment of their cognate coreceptor. (B) BaL or JRFL pseudoviruses were bound to TZM-bl cells in the cold and induced to fuse by shifting to 37°C. The fusion was stopped at indicated times either by adding 1 µM C52L or by briefly placing cells on ice. Cells were then loaded with the BlaM substrate and incubated overnight at 13.5°C, a temperature that prevented HIV fusion but allowed the cleavage of the BlaM substrate.
(0.46 MB TIF)

Figure S4 Simulation of the effect of changes in k_2_ and k_i_ on the fusion kinetics. (A) A model of HIV fusion in which the equality requirement for the inactivation rate constants at different stages of fusion has been relaxed. Thus, in addition to k_1_, k_2_ and k_3_ constants, the reaction is described by three inactivation rate constants, k_i0_, k_i1_ and k_i2_. (B) Modeling the fusion kinetics using the rate constants similar to those obtained for V3BaL fusion (see Table 1). (C, D) Changes in the kinetics of the escape from C-peptides due to an 8-fold increase in k_3_ or k_i2_, respectively.
(0.66 MB TIF)

Appendix S1 (0.08 MB DOC)
