# Supplementary figures and images for: Early Steps of HIV-1 Fusion Define the Sensitivity to Inhibitory Peptides That Block 6-Helix Bundle Formation
Source: PLoS Pathog. 2009 Sep 18;5(9):e1000585. doi: 10.1371/journal.ppat.1000585 (PMC2736578; doi:10.1371/journal.ppat.1000585)

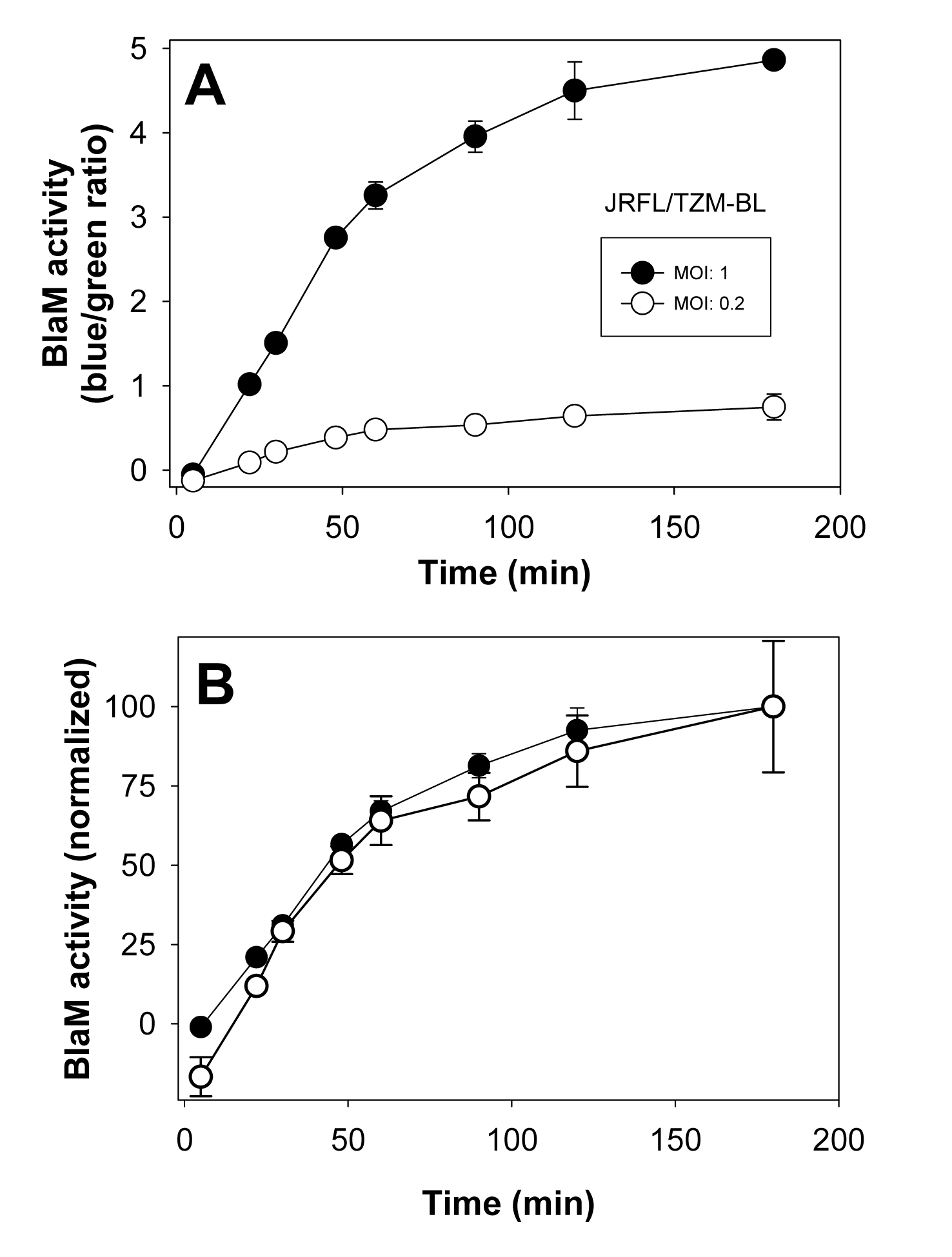

Supplement: Figure S1 — The normalized kinetics of fusion is independent of the number of cell-bound viruses. JRFL viruses were added to TZM-bl cells at the multiplicity of infection (MOI) 1 or 0.2. After pre-binding the viruses at 4°C, cells were washed and shifted to 37°C to induce fusion. (A) The kinetics of BlaM signal for the two different MOI is shown. (B) The kinetics of fusion after normalizing the data to the last time point. Even though the overall BlaM signal is proportional to the MOI, the rate of fusion is independent of the virus input. (0.37 MB TIF) [file ppat.1000585.s001.tif]

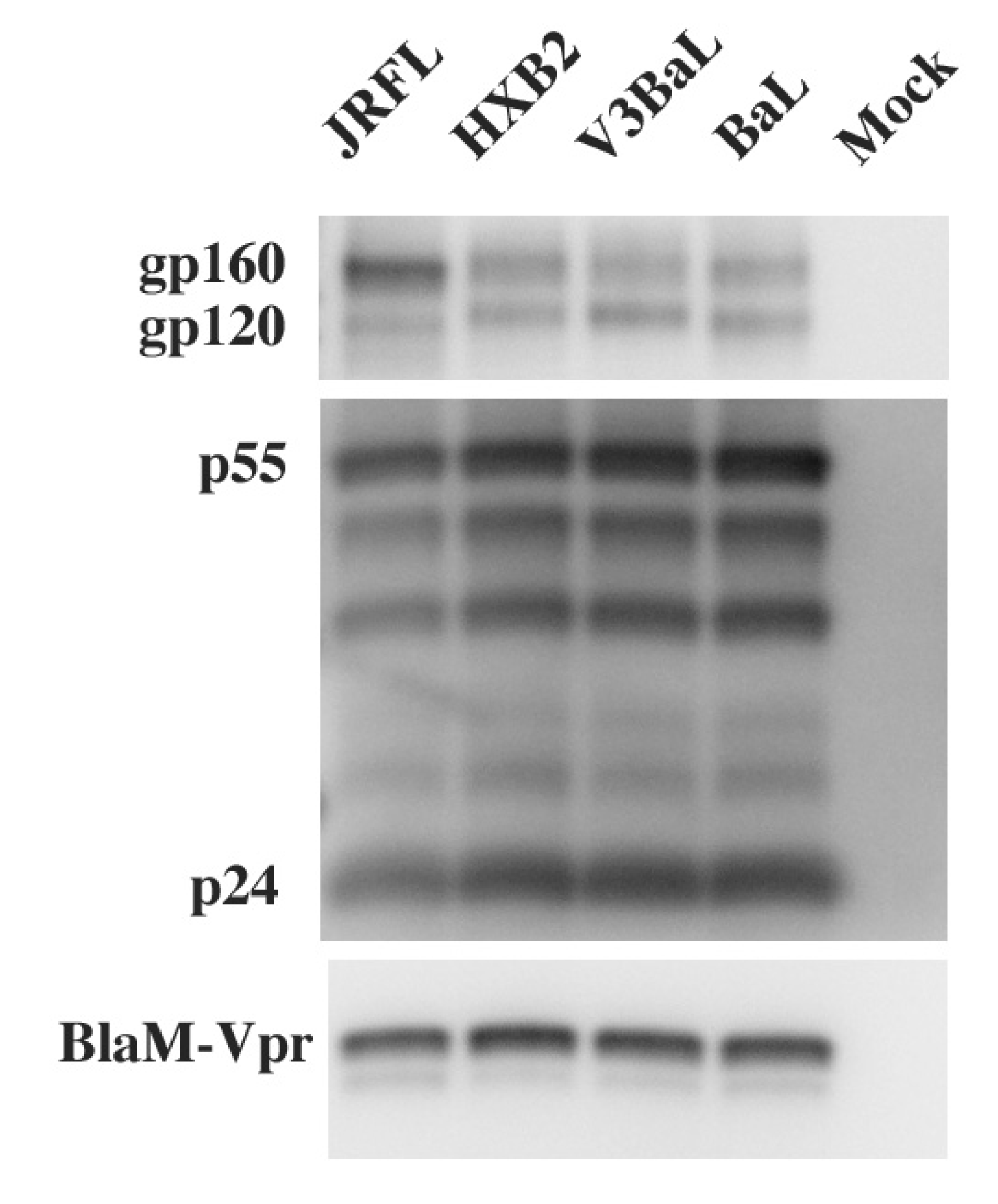

Supplement: Figure S2 — Processing and incorporation of various HIV-1 Env glycoproteins into pseudoviruses assessed by Western blotting. Viral lysates were prepared with RIPA lysis buffer (0.05 M TrisCl, 0.15 M NaCl, 1% Triton X-100, 0.1% sodium dodecyl sulfate (SDS) and 1% sodium deoxycholate) that contained the Complete protease inhibitor (Roche Applied Science, Mannheim, Germany). The samples were separated on a 10% SDS-PAGE gel and transferred to PVDF membrane (Millipore, Billerica, MA). The viral proteins were then detected with anti-β-lactamase monoclonal antibody (QED Bioscience Inc, San Diego, CA), anti-p24 sera (from AIDS Research and Reference Reagent Program), and anti-gp120 polyclonal antibody (Fitzgerald, Concord, MA). The blot was treated with biotinylated anti-mouse or rabbit immunoglobulin (GE Healthcare Bio-Sciences AB, Uppsala, Sweden), or anti-goat immunoglobulin (Sigma) as the secondary antibody and then incubated with streptavidin-horseradish peroxidase conjugate (GE Healthcare Bio-Sciences). Chemiluminescence from the protein bands was visualized on the Fluor Chem SP (Alpha Innotech, San Leandro, CA) using the Lumi-Lightplus (Roche) substrate. (1.04 MB TIF) [file ppat.1000585.s002.tif]

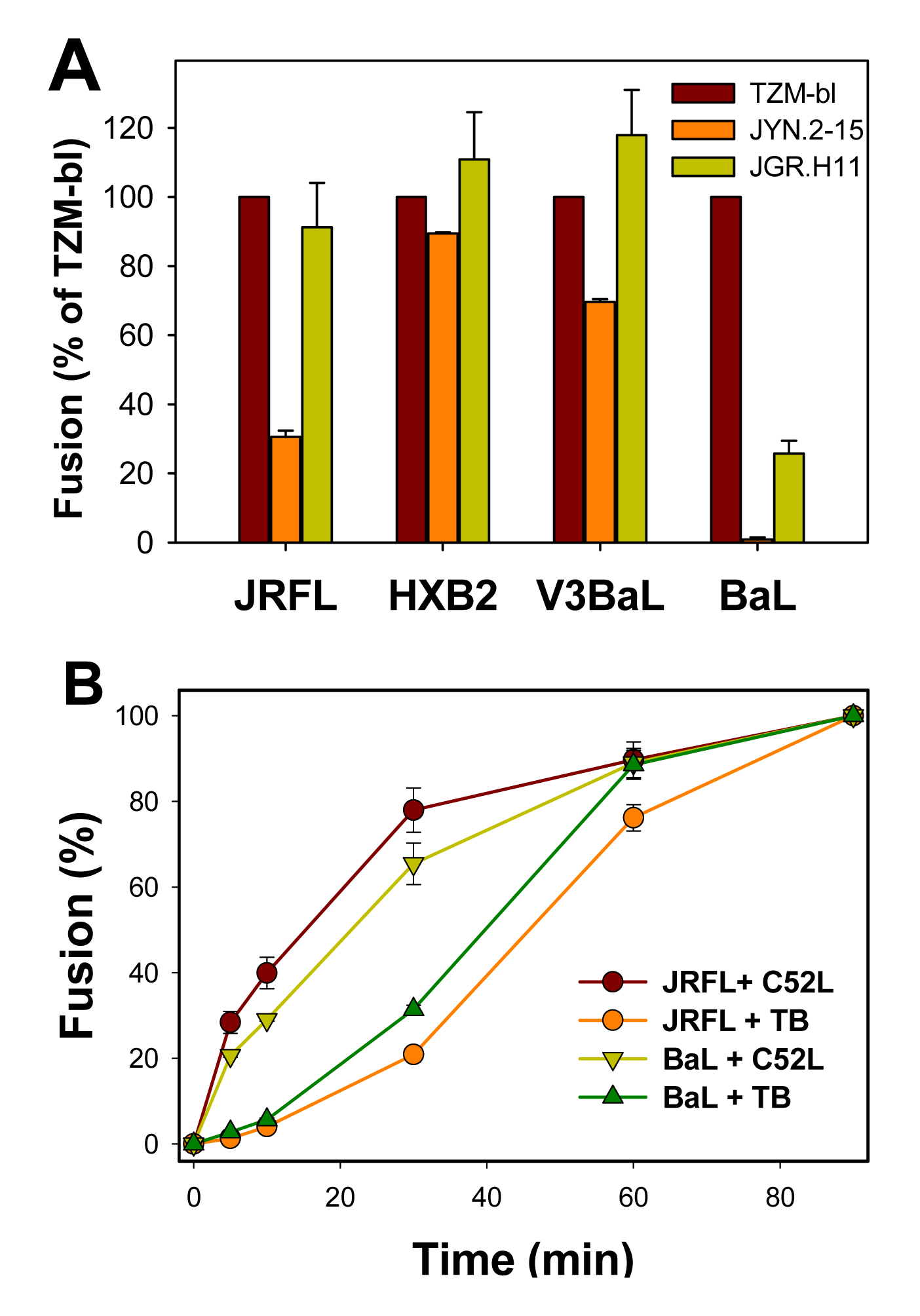

Supplement: Figure S3 — Fusion with target cells expressing mutant CCR5 and escape from C52L and the temperature block. (A) The efficacy of JRFL, HXB2, BaL and V3BaL fusion with HeLa cells expressing similar levels of CD4, CXCR4 and wild-type (TZM-bl) or mutant (JYN.2-15 and JGR.H11) CCR5. In these experiments, the viral input was normalized by inoculating the cells with the same amount of virus (an equivalent of MOI 0.7 for TZM-bl cells). Note that BaL failed to fuse with cells expressing the Y14N CCR5 and fused inefficiently with cells expressing the G163R mutant. The ability of V3BaL to utilize the Y14N CCR5 mutant is consistent with the less pronounced dependency of X4-tropic viruses on the N-terminal segment of their cognate coreceptor. (B) BaL or JRFL pseudoviruses were bound to TZM-bl cells in the cold and induced to fuse by shifting to 37°C. The fusion was stopped at indicated times either by adding 1 µM C52L or by briefly placing cells on ice. Cells were then loaded with the BlaM substrate and incubated overnight at 13.5°C, a temperature that prevented HIV fusion but allowed the cleavage of the BlaM substrate. (0.46 MB TIF) [file ppat.1000585.s003.tif]

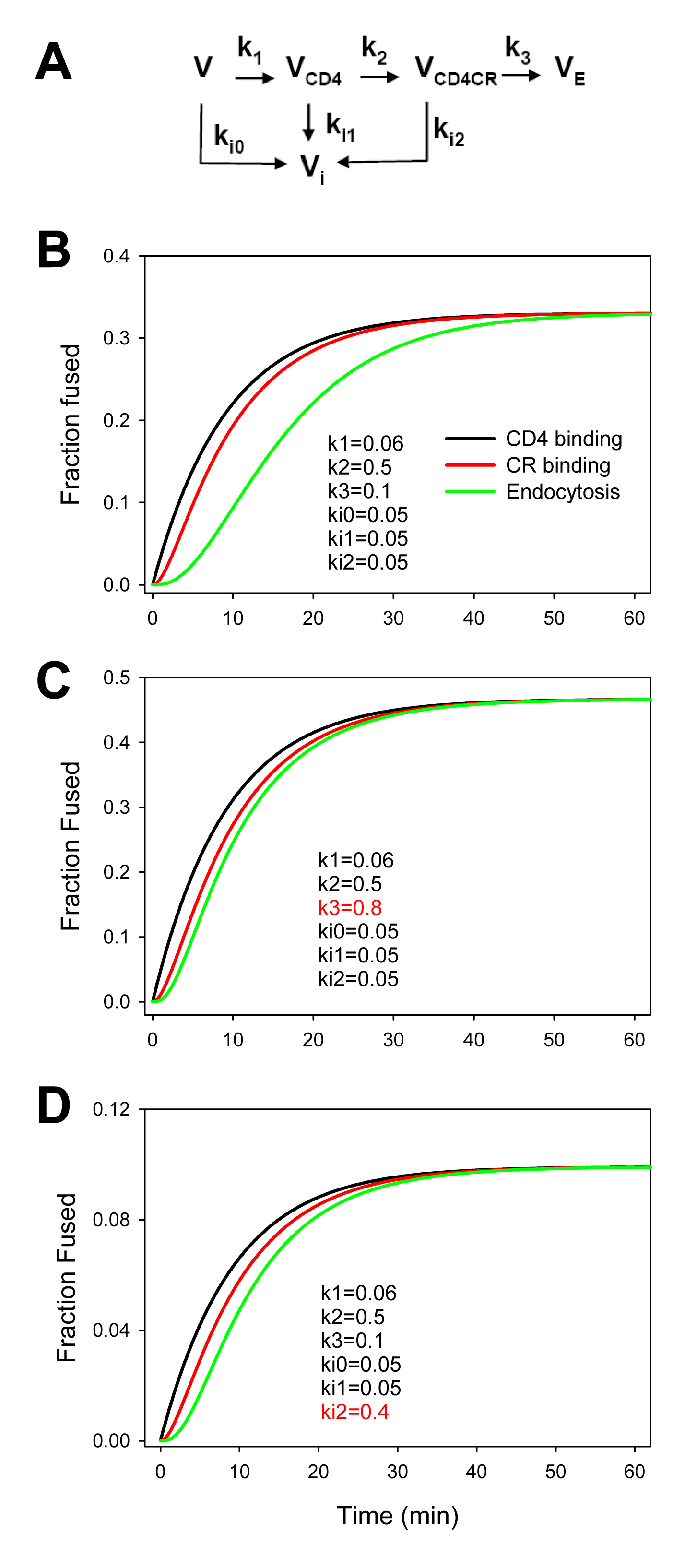

Supplement: Figure S4 — Simulation of the effect of changes in k2 and ki on the fusion kinetics. (A) A model of HIV fusion in which the equality requirement for the inactivation rate constants at different stages of fusion has been relaxed. Thus, in addition to k1, k2 and k3 constants, the reaction is described by three inactivation rate constants, ki0, ki1 and ki2. (B) Modeling the fusion kinetics using the rate constants similar to those obtained for V3BaL fusion (see Table 1). (C, D) Changes in the kinetics of the escape from C-peptides due to an 8-fold increase in k3 or ki2, respectively. (0.66 MB TIF) [file ppat.1000585.s004.tif]
