# Supplementary material for: Early Steps of HIV-1 Fusion Define the Sensitivity to Inhibitory Peptides That Block 6-Helix Bundle Formation
Source: PLoS Pathog. 2009 Sep 18;5(9):e1000585. doi: 10.1371/journal.ppat.1000585 (PMC2736578; doi:10.1371/journal.ppat.1000585)
Supplement: Appendix S1 — (0.08 MB DOC) [file ppat.1000585.s005.doc]

**APPENDIX**

**I. Kinetic model of HIV-cell fusion.**

The simplified kinetic scheme for HIV fusion corresponding to virus progression through four states (see the main text for definitions and Fig. 1C for illustration) can be presented as:

k_1_ k_2_ k_3_

V 🡪 V_CD4_ 🡪 V_CD4CR_ 🡪 V_E_ 🡪 … 🡪 V_F_

k_i_

V_I_

Using the notations introduced in the main text, the time evolution of the system according to this kinetic scheme is described by the following differential equations for the amounts of viruses in each of the states:

$\frac{dV}{dt}=-(k_{1}+k_{i})\cdot V$ (A1)

(A2)

(A3)

(A4)

The solution of these equations gives:

$V=V_{tot}\cdot$ $\exp[-(k_{1}+k_{i})t]$ (A5)

$V_{CD4}=\frac{k_{1}\cdot V_{tot}}{(k_{2}-k_{1})}\cdot\left\{\exp[-(k_{1}+k_{i})t]-\exp[-(k_{2}+k_{i})t]\right\}$ (A6)

$\begin{matrix} V_{CD4CR}=\frac{k_{1}\cdot k_{2}\cdot V_{tot}}{(k_{2}-k_{1})(k_{3}-k_{1})}\cdot\left\{\exp[-(k_{1}+k_{i})t]-\exp[-(k_{3}+k_{i})t]\right\} \\ -\frac{k_{1}\cdot k_{2}\cdot V_{tot}}{(k_{2}-k_{1})(k_{3}-k_{2})}\cdot\left\{\exp[-(k_{2}+k_{i})t]-\exp[-(k_{3}+k_{i})t]\right\} \end{matrix}$ (A7)

$\begin{matrix} V_{E}=V_{ext}-\frac{k_{1}\cdot k_{2}\cdot k_{3}\cdot V_{tot}}{(k_{2}-k_{1})(k_{3}-k_{1})(k_{1}+k_{i})}\cdot\exp[-(k_{1}+k_{i})t] \\ +\frac{k_{1}\cdot k_{2}\cdot k_{3}\cdot V_{tot}}{(k_{2}-k_{1})(k_{3}-k_{2})(k_{2}+k_{i})}\cdot\exp[-(k_{2}+k_{i})t]-\frac{k_{1}\cdot k_{2}\cdot k_{3}\cdot V_{tot}}{(k_{3}+k_{i})(k_{3}-k_{1})(k_{3}-k_{2})}\cdot\exp[-(k_{3}+k_{i})t] \end{matrix}$(A8)

where V_tot_ is the total number of the fusion-competent viral particles bound to cells at time = 0; V_ext_ is the total number of viruses fused at an infinitely long time, which is given by:

$V_{ext}=\frac{k_{1}\cdot k_{2}\cdot k_{3}\cdot V_{tot}}{(k_{3}+k_{i})(k_{1}+k_{i})(k_{2}+k_{i})}$ (A9)

**II. Description of the time-of-addition experiments using CD4 and coreceptor binding inhibitors.**

Analogously to the previous section, the kinetic equations can be readily derived for the virus progression along the pathway (Fig. 1C) in case where inhibitors of the CD4 or co-receptor binding are added at varied times of virus-cell incubation. Solution of these equations gives the time dependence of the amounts of viruses V_CD4_* and V_CD4CR_* which undergo productive endocytosis upon addition of inhibitors of CD4 or co-receptor binding, respectively. Denoting by t* the time point of the inhibitor addition and by *τ = t – t** the time of the system evolution in the presence of the inhibitor, we obtain:

(A10)

(A11)

Where:

$$\begin{matrix} V_{CD4CR}(t^{*})=\frac{k_{1}\cdot k_{2}\cdot V_{tot}}{(k_{2}-k_{1})(k_{3}-k_{1})}\cdot\left\{\exp[-(k_{1}+k_{i})t^{*}]-\exp[-(k_{3}+k_{i})t^{*}]\right\} \\ -\frac{k_{1}\cdot k_{2}\cdot V_{tot}}{(k_{2}-k_{1})(k_{3}-k_{2})}\cdot\left\{\exp[-(k_{2}+k_{i})t^{*}]-\exp[-(k_{3}+k_{i})t^{*}]\right\} \end{matrix}$$

(A12)

**III. Time averages of fusion intermediates.**

The time averages (over the time interval from 0 to *t***) of *V_CD4_* and *V_CD4CR_* can be obtained by integrating equations (6) and (7) and normalizing over the total time of the experiment, t**:

$<V_{CD4}>=\frac{1}{t^{\text{**}}}\frac{k_{1}\cdot V_{tot}}{(k_{2}-k_{1})}\cdot\left\{\frac{1}{k_{1}+k_{i}}[1-\exp(-(k_{1}+k_{i})t^{**})]-\frac{1}{k_{2}+k_{i}}[1-\exp(-(k_{2}+k_{i})t^{**})]\right\}$(A13)

$\begin{matrix} <V_{CD4CR}>=\frac{1}{t^{**}}\frac{k_{1}\cdot k_{2}\cdot V_{tot}}{(k_{2}-k_{1})(k_{3}-k_{1})}\cdot\left\{\begin{matrix} \frac{1}{k_{1}+k_{i}}[1-\exp(-(k_{1}+k_{i})t^{**})]- \\ -\frac{1}{k_{3}+k_{i}}[1-\exp(-(k_{3}+k_{i})t^{**})] \end{matrix}\right\} \\ -\frac{1}{t^{**}}\frac{k_{1}\cdot k_{2}\cdot V_{tot}}{(k_{2}-k_{1})(k_{3}-k_{2})}\cdot\left\{\begin{matrix} \frac{1}{k_{2}+k_{i}}[1-\exp(-(k_{2}+k_{i})t^{**})]- \\ -\frac{1}{k_{F}+k_{i}}[1-\exp(-(k_{3}+k_{i})t^{**})] \end{matrix}\right\} \end{matrix}$ (A14)
